# Temperament-based treatment for young adults with eating disorders: acceptability and initial efficacy of an intensive, multi-family, parent-involved treatment

**DOI:** 10.1186/s40337-021-00465-x

**Published:** 2021-09-08

**Authors:** Stephanie Knatz Peck, Terra Towne, Christina E. Wierenga, Laura Hill, Ivan Eisler, Tiffany Brown, Emily Han, McKenzie Miller, Taylor Perry, Walter Kaye

**Affiliations:** 1 Eating Disorder Treatment & Research Center, Department of Psychiatry, University of California, San Diego, 4510 Executive Drive Suite 315, San Diego, CA 92121 USA; 2 King’s College London, London, UK; 3 Maudsley Centre for Child Adolescent Eating Disorders, London, UK

**Keywords:** Eating disorders, Psychological treatment, Treatment, Young adults, Anorexia nervosa, Bulimia nervosa, Avoidant restrictive food intake disorder, Family-based treatment

## Abstract

**Background:**

Adult eating disorder treatments are hampered by lack of access and limited efficacy. This open-trial study evaluated the acceptability and preliminary efficacy of a novel intervention for adults with eating disorders delivered to young adults and parent-supports in an intensive, multi-family format (Young Adult Temperament-Based Treatment with Supports; YA-TBT-S).

**Methods:**

38 YA-TBT-S participants (*m* age = 19.58; SD 2.13) with anorexia nervosa (AN)-spectrum disorders, bulimia nervosa (BN)-spectrum disorders, and avoidant/restrictive food intake disorder (ARFID) completed self-report assessments at admission, discharge, and 12-month follow-up. Assessments measured program satisfaction, eating disorder psychopathology and impairment, body mass index (BMI), and trait anxiety. Outcomes were analyzed using linear mixed effects models to examine changes in outcome variables over time.

**Results:**

Treatment was rated as highly satisfactory. 53.33% were in partial or full remission at 12-month follow-up. 56% of participants received other treatment within the 12-month follow-up period, suggesting that YA-TBT-S may be an adjunctive treatment. Participants reported reductions in ED symptomatology (AN and BN), increases in BMI (AN and ARFID), and reductions in clinical impairment (AN and ARFID) at 12-month follow-up.

**Conclusions:**

YA-TBT-S is a feasible and acceptable adjunctive treatment for young adults with a broad range of ED diagnoses and may be a method for involving parents in ED treatment in ways that are acceptable to both parents and YA. Further evaluation of efficacy is needed in larger samples, and to compare YA-TBT-S to other ED treatment approaches.

**Plain English summary** Eating disorders are costly and dangerous psychiatric disorders that affect millions of individuals each year. Despite their risks and societal costs, currently available treatments are limited. This study examined the acceptability and efficacy of Young Adult, Temperament-Based Treatment with Supports (YA-TBT-S), a new treatment program for adults with eating disorders. YA-TBT-S was rated highly, and a significant portion of participants improved based on ratings collected 12 months after program participation. Those with anorexia nervosa (AN) and bulimia nervosa (BN) showed significant reductions in eating disorder pathology, and those with AN and avoidant/restrictive food intake disorder (ARFID) showed increases in BMI over time.

## Introduction

Eating disorders (ED), including anorexia (AN), bulimia (BN), and avoidant/restrictive food intake disorder (ARFID) are costly and dangerous mental illnesses with high morbidity and mortality [1–4]. These illnesses are amongst the most common causes of disability in young women in high income nations and are associated with significant personal, economic, and societal burden [5]. Despite these burdens and risks, there are significant health care utilization issues, and these disorders frequently go under-treated [6]. As such, improving accessible treatments of EDs is of immense clinical and public health importance due to their danger and disease burden.

In addition to limitations regarding accessibility, currently available treatments have limited efficacy for adults. With regards to AN, there are currently no treatments that have demonstrated primacy. Studies suggest that treatment effects are relatively modest and drop-out rates are high for many first-line behavioral treatments. Even for those who receive the most efficacious outpatient treatments, about 50–60% of patients do not achieve weight restoration and psychological remission [7–11]. There is an urgent need to develop and improve therapies for this population due to their lethality, high relapse rates, and economic costs [12].

For BN, a number of specialized psychological interventions have been shown to be efficacious. However, treatment effects are limited [13–15]. A recent meta-analysis of psychological treatments for BN showed that over 50% of treatment participants continue to engage in ED behaviors following treatment [16]. There are not currently any empirically-supported treatments for adults with avoidant/restrictive food intake disorder although CBT for ARFID is promising [17]. Accordingly, even the most well-studied treatment approaches are limited.

Novel models of treatment are needed to address these limitations and improve access to care. Considerations for new treatment models include: (1) treatments targeting specific population subsets that are disproportionately affected, in our case, young adults; (2) parent-supported models of treatment based on a contextual and developmental understanding of parent-YA relationships; (3) a mechanistic understanding of EDs based on contemporary studies and new insights about the underlying neurobiology; and (4) novel treatment formats that span beyond the current paradigms of weekly outpatient psychotherapy and/or traditional long-term intensive care (inpatient, residential, and partial hospitalization programs).

### Treatments for YA

There is a critical need to develop and improve ED treatments for young adults (YA). EDs most commonly emerge and remain prevalent during young adulthood [18–20]. Studies conducted in college populations confirm that EDs are prevalent and persistent [21].

ED treatments specifically targeting YA and informed by developmental stage could improve treatment outcomes in line with a personalized medicine approach. Effective age-adapted ED treatments exist for children and adolescents [7, 8]. yet few developmentally-informed treatments are available for YA. The most commonly used first-line interventions for YA with EDs are not designed within a developmental framework. In response to the critical need to address this large subset of the ED population in a more focused manner, YA-specific treatments have emerged, and preliminary results appear promising. These include FBT adapted for transition-aged youth (FBT-TAY; FBTY) and the treatments utilized at the First Episode Rapid Early Intervention Program (FREED) [22–25]. However, more research is needed to continue to develop and test YA-specific treatments.

Much like adolescence, young adulthood has been validated as a distinctive developmental stage, known as “emerging adulthood” with specific developmental milestones and tasks [26, 27]. This life stage includes important characteristics that distinguish those in this age class from both adolescents and older adults. Assisting healthy growth through this stage, including recovery from an ED, requires an understanding of both the YA developmental need for autonomy and independence and their inter-dependence on their family of origin, including parents. Developing and disseminating specific treatments accounting for these factors and targeted to YA with EDs is important and may improve treatment utilization and outcomes and reduce lifetime disease burden and the risk of a chronic course later in life.

### Parent-supported models of treatment

Increasingly so, YA receive financial, living, and other types of support from their family of origin [28, 29]. Despite the familial interdependence that often defines the YA life stage, parents and families are not traditionally involved in adult ED treatment. Since EDs most commonly occur during young adulthood, more family-involved models of treatment that prescribe developmentally-appropriate parent support are needed for YA. Recent efforts to adapt conventional outpatient FBT to young adults (FBTY, FBT-TAY) [30, 31] and to tailor treatments to transitional age youth by involving family [32] are promising, with large effects for weight gain and symptom reduction at end of treatment. Similarly, the FREED program’s utilization of the Maudsley Anorexia Nervosa Treatment for Adults (MANTRA) for YA includes developmentally appropriate family involvement, but more research is needed [25].

### Treatments addressing temperament and neurobiology

Treatments that are based on an updated mechanistic understanding of ED behavior have the potential to improve treatment outcomes [12]. Contemporary research has shed light on altered neural circuity, and related temperament and personality features that confer risk and contribute to the development of an ED [33, 34]. We developed a temperament-based treatment approach for EDs (Temperament-Based Treatment with Supports, TBT-S) that is structured for common ED temperament and personality traits [35]. This approach provides psychoeducation on the role of biology and temperament in eating disorders with the aim of reducing blame and increasing understanding. Notably, Zhou et al. [36] found that providing psychoeducation on the role of genes and the effect of EDs on the brain had a significant impact on ED psychopathology and body acceptance. Additionally, TBT-S targets underlying mechanisms at play in disordered eating behavior [37–39].

### Advantages of intensive treatments

The majority of rigorous trials have evaluated outpatient treatment modalities intended to be delivered in weekly, hour-long sessions. As treatments for EDs, and mental illness more generally, have stagnated in their progress, novel treatment paradigms testing other formats for treatment delivery should be evaluated. New paradigms for providing treatment may increase access and have the potential to improve outcomes. We developed a 5-day intensive, multi-family therapy approach in which participants receive approximately 40 h of treatment over the course of five days. This intensive treatment approach has demonstrated good feasibility, acceptability, and outcomes with adolescents and adults with EDs and has been described in detail elsewhere [40, 41]. The intensive format leverages aspects of traditional long-term intensive treatment programs that may contribute to behavior change, such as structure and monitored meal practice. In this model, parents attend with YA to receive psychoeducation and skills training and practice and learn developmentally appropriate methods of support. The treatment is delivered in a multi-family format, allowing participants to benefit from peer-to-peer consultation and build a support network, which can improve outcomes [42, 43]. Multi Family Therapy groups have been successfully used in populations of adolescents and young adults with EDs [44, 45].

### Study aims and hypotheses

The present study sought to examine the acceptability, feasibility, and preliminary treatment outcomes for program attendees of a model of TBT-S adapted for YA, using a naturalistic design. It aimed to expand upon Wierenga and colleagues’ examination of TBT-S [37] by reporting outcomes for a mixed diagnostic sample that is reflective of the program’s patient population at 12-month follow-up. This expansion allows for the evaluation of symptom trajectory and recovery rates among a heterogeneous range of patients presenting for TBT-S over a longer period of follow-up given the high rates of relapse upon discharging from higher levels of care [16]. Additionally, outcome analyses in the present study accounted for missing data to address a potential response bias in the previous study.

Our hypotheses were as follows: (a) the program would be highly acceptable to participants; (b) participants would demonstrate significant decreases in ED symptoms across all diagnoses from admission through follow-up; (c) AN and ARFID participants would demonstrate significant increases in BMI from admission through follow-up; (d) remission rates would be comparable to those in other adult ED treatment studies; and (e) patients would experience clinically meaningful reductions in anxiety due to the focus on treating anxiety as a mechanistic target.

## Methods

### Participants and procedure

Data for the present study came from 38 young adults who were admitted to the University of California San Diego (UCSD), Young Adult Intensive Family Treatment Program (YA-IFT) between October 2017 and June 2019. Patients who sought out the program were self-referred or referred by other healthcare providers. Criteria for admission to the program included a primary ED diagnosis, attendance with at least one designated primary support person, and medical stability assessed through review of relevant medical information by UCSD program physicians. Participants met criteria for an ED based on the *Diagnostic and Statistical Manual of Mental Disorders- Fifth Edition* [46]. Diagnoses were made by trained raters administering the Structured Clinical Interview (SCID) for DSM-5 [47]. For patients who did not complete the SCID (n = 8), diagnoses were ascertained by staff psychiatrists using a semi-structured interview. For the purposes of this study, patients classified with an AN diagnosis included AN-spectrum disorders: AN-R (n = 12), AN-BP (n = 7), atypical AN (n = 4), and subthreshold AN (n = 1). AN spectrum disorders included both patients who are not in remission and who are in partial remission. For diagnostic classification purposes, partial remission for anorexia nervosa was defined as 1) meeting DSM diagnostic criteria for either anorexia nervosa restricting type or binge-purge type with the exception of admit BMI being ≥ 18.5 and 2) BMI being ≤ 18.5 within the past year. Nine of the 23 patients with AN-spectrum disorders had BMIs less than 18.5.

Patients classified with BN (n = 8) included one patient with subthreshold BN. Six patients diagnosed with ARFID participated.

The study used a naturalistic design and participants who consented to participate in the study completed a structured clinical interview assessing for DSM-5 diagnoses upon admission and online self-report questionnaires at admission, discharge, and at 12-month follow-up. Participants were compensated with a $40 gift card for survey completion at 12-month follow-up. All study procedures were approved by the UCSD Institutional Review Board.

### TBT-S program description

YA-TBT-S is a short-term, intensive treatment specifically designed for YA and their parent(s). YA-TBT-S was delivered in a five-day, intensive, multi-family format. The treatment introduces a model of parent involvement that is tailored to developmental stage and addresses eating behavior from a framework of temperament and neurobiology. Additionally, participants received: psychoeducation on temperament and neurobiology; experiential activities focused on explaining neurobiology and building effective YA-parent relationships; skills training; dietary support; and in-vivo, therapist-assisted coaching during 21 therapeutic meals and snacks.

### Outcome measures

#### Study acceptability

An acceptability measure was designed to assess satisfaction. The measure consisted of 22 items assessing degree of satisfaction with the overall program, specific components of the treatment including neurobiology exercises and education, parent involvement, participants’ perceptions of feeling equipped to recover, and multi-family group support. Participants rated satisfaction on a 5-point Likert scale (1 = Strongly Disagree to 5 = Strongly Agree) and completed the survey at post-treatment. Cronbach’s alpha was 0.92.

#### Study adherence

Questions were devised to assess adherence to specific treatment recommendations that are considered unique and fundamental to the treatment program, including neurobiology activities and education, parent involvement, and behavioral contracting. Participants self-reported adherence to specific treatment components at 12-month follow-up by reporting on the frequency of use based on a 5-point scale ranging from *Never* to *Always.*

#### Client satisfaction questionnaire (CSQ-8)

The present study used an 8-item version of the CSQ to assess satisfaction with treatment length, quality, and delivery. Higher scores indicate greater satisfaction, with scores above 26 indicating high levels of satisfaction. Patients completed the CSQ at post-treatment. Cronbach alpha was 0.74 [48].

#### Body mass index

Height and weight were measured at admission and discharge (height at admission only) by a staff nurse and were used to calculate BMI (kg/m^2^). Self-reported weight was used to calculate BMI at follow-up. Measured weight and self-reported weight at baseline were strongly positively correlated, *r* (25) = 0.98, *p* =  < 0.001, providing support for the use of self-reported data at follow-up.

#### Eating disorder examination questionnaire

The Eating Disorder Examination Questionnaire (EDE-Q; Fairburn & Beglin, 1994) is a 31-item self-report questionnaire used to evaluate the presence and severity of eating pathology during the previous 28 days. The EDE-Q is a widely used measure with good validity and reliability. The scale renders a composite score, the Global EDE-Q score, which was used in the present study as the measure of general ED pathology [49]. The measure was modified at discharge to assess for eating pathology over the past seven days due to the short-term nature of the program. Response items were re-scaled to converge with the seven-day time course (0 = no days to 6 = every day). The EDE-QS, a short-form version of the EDE-Q with a modified response scale to assess change over a shorter time course, has demonstrated good reliability and validity, and performs similarly to the EDE-Q [50]. Cronbach’s Alpha for Global EDE-Q was 0.95. The EDE-Q also assesses counts of eating disorder behaviors, which were used in the present study to assess bingeing and purging frequencies. In order to compare across all time points, discharge scores for items measuring frequency of binge and purge behaviors were then multiplied by four to ensure the time period assessed was equivalent to that of pre-treatment and follow-up.

#### Clinical Impairment Assessment (CIA)

The CIA is a 16-item self-report measure of the severity of psychosocial impairment due to ED features in the past 28 days [51]. Like the EDEQ, the CIA was modified at discharge to reflect the past seven days. The measure assesses impairment in primary domains based on eating pathology. Cronbach’s alpha was 0.98.

#### State-Trait Anxiety Inventory- Trait subscale

The State-Trait Anxiety Inventory- Trait subscale (STAI-T) [52] is a 20-item self-report measure assessing the presence of trait anxiety. Items were modified at discharge to reflect the past seven days. Cronbach’s Alpha was 0.98.

#### Remission criteria

The recovery criteria established by Bardone-Cone and colleagues were used to classify remission status among participants with AN and BN [53]. Specifically, full remission was rigorously defined as having a BMI greater than 18.5 kg/m^2^, no binge eating or purging for the past 28 days, and an EDE-Q Global score less than 1 SD above the mean for young adults ages 18 to 22 (i.e., 2.91) [54]. The partial remission criteria were the same as the full remission criteria with the exception of an elevated EDE-Q Global score. Because ARFID-specific measures were not included in the study, participants with ARFID were classified dichotomously (i.e., remitted or not remitted) based on their BMI and CIA scores. ARFID remission was defined as having a BMI above 18.5 kg/m^2^ and a CIA score below the clinical cut-point. Remissions rates were reported as a percentage of the total sample, as remission status of participants who did not complete surveys could not always be discerned. Participants who did not complete admission or discharge surveys but were objectively underweight at these time points were classified as not remitted.

### Statistical analyses

Independent samples t-tests and chi square tests were used to examine patterns of missing data and predictors of loss to follow-up. McNemar exact tests examined differences in both the proportion of participants who were hospitalized pre- and post-YA-TBT-S and the proportion of participants reporting a good or poor outcome at admission, discharge, and follow-up.

Linear mixed models were used to evaluate changes in continuous outcomes variables over time (admission to follow-up). All participants, including those who dropped out of YA-TBT-S, were included in analyses. To account for missing data, full information maximum likelihood estimation was used. Repeated measurements of dependent variables (EDE-Q Global, CIA, bingeing, purging, and STAI) nested within participants were modeled at level 1. Given variability in the timing of follow-up assessments and the aim of examining symptomatology over time, time was modeled continuously and centered at YA-TBT-S admission (admission = day 0). To account for participants being nested within treatment weeks, YA-TBT-S week and the interaction between YA-TBT-S week and time were modeled at level 2. Age and length of illness were included as covariates in all models.

Model fitting was conducted in stages, and subsequent models were evaluated using log likelihood, Akaike’s information criterion, and Bayesian information criteria. Quadratic terms (i.e., time squared and time cubed) did not improve model fit and were removed from final models. Final EDE-Q Global and STAI trait models represent random intercept, fixed slope models, while the final CIA model was modeled as a random intercept, random slope model. All participants, regardless of diagnosis, were included in the STAI and CIA models. BMI and EDE-Q Global models examined subsets of participants based on the relevance of these outcome variables to their ED diagnosis. Specifically, low weight participants (i.e., BMI less than 18.5 kg/m^2^) with AN and ARFID were included in the BMI model, and participants with AN and BN were included in the EDE-Q Global model. Correlation coefficients describing the magnitude of relationships between predictors and dependent variables in the multilevel models were calculated by taking the square root of the following equation: *t*^2^/(*t*^2^ + *df*).

To examine changes in binge eating and purging frequencies over time, generalized linear mixed models fit with a Poisson distribution and log link function were attempted. Despite attempts to revise the fit of the models, model convergence issues (i.e., the final Hessian matrix not being positive definite) precluded the use of multilevel modeling to measure changes in these behaviors over time. As such, mean bingeing and purging frequencies at admission, discharge, and follow-up were reported among participants with AN-BP and BN.

To approximate the number of subjects needed to adequately power our multilevel models, a power analysis was conducted for a mixed repeated measures Anova with six groups (i.e., the number of separate treatment groups) and three repeated measurements (i.e., admission, discharge, and follow-up). To detect a medium size effect with 80% power, 54 subjects were needed, suggesting the present study was likely underpowered to examine nuanced interaction effects.

## Results

### Patient demographics

Table 1 presents demographic data across diagnoses. The average age of the sample was approximately 19 years across all diagnoses. ARFID cases had the longest duration of illness at 6.96 years. BN patients had an average illness duration of 4.01 years, whereas AN patients in this sample had a relatively shorter duration of illness of 2.21 years. 37.5% of AN cases and 100% of ARFID cases were considered underweight with BMIs below 18.5. There were significant comorbidities across diagnoses, with 20–60% of patients exhibiting comorbid anxiety and mood disorders. The sample was predominantly White and cisgender female. Participants reported a bimodal household income of greater than $250,000 (16.67%) and less than $10,000 (16.67%) per year. Twenty-one percent of participants reported annual household incomes below $20,000, 20.8% reported household incomes between $70,001 and $100,000, and 29.2% reported household incomes between $100,001 and $210,000. It is unclear if participants accounted for financial support received from their families. Prior to admission, participants rated the suitability of therapy for their problem as an average of 7.82/10 (SD = 2.04) and the anticipated success of therapy as a 6.73/10 (SD = 2.51).

**Table 1:** Descriptive information of sample by diagnosis

	AN (*n* = 24)	BN (*n* = 8)	ARFID (*n* = 6)
	*M* (SD)/*n* (%)	*M* (SD)/*n* (%)	*M* (SD)/*n* (%)
Age	19.67 (2.22)	19.75 (2.49)	19.92 (1.27)
Illness duration (years)	2.21 (1.77)	4.01 (1.93)	6.96 (9.37)
BMI (kg/m^2^)	19.94 (2.82)	22.11 (1.13)	17.23 (1.11)
BMI < 18.5	9 (37.5%)	0	6 (100%)
% Female	91.7%	100%	83.3%
Ethnicity			
White	19 (79.2%)	7 (87.5%)	5 (83.3%)
Hispanic	1 (4.2%)	1 (4.2%)	-
Asian	2 (8.3%)	–	1 (16.7%)
Native American	1 (4.2%)	–	–
Other	1 (4.2%)	–	–

% with co-morbid diagnoses	*n* = 19	*n* = 6	*n* = 6
Mood disorder	6 (31.6%)	3 (50%)	2 (33.3%)
Anxiety disorder	5 (20.9%)	3 (50%)	6 (66.7%)
Substance use disorder	1 (4.2%)	2 (25%)	1 (16.7%)
Trauma-related disorder	1 (4.2%)	1 (12.5%)	1 (16.7%)
Other co-morbid disorder	1 (20.8%)	2 (25%)	1 (16.7%)

### Patient retention & predictors of loss to follow-up

**Fig. 1:**
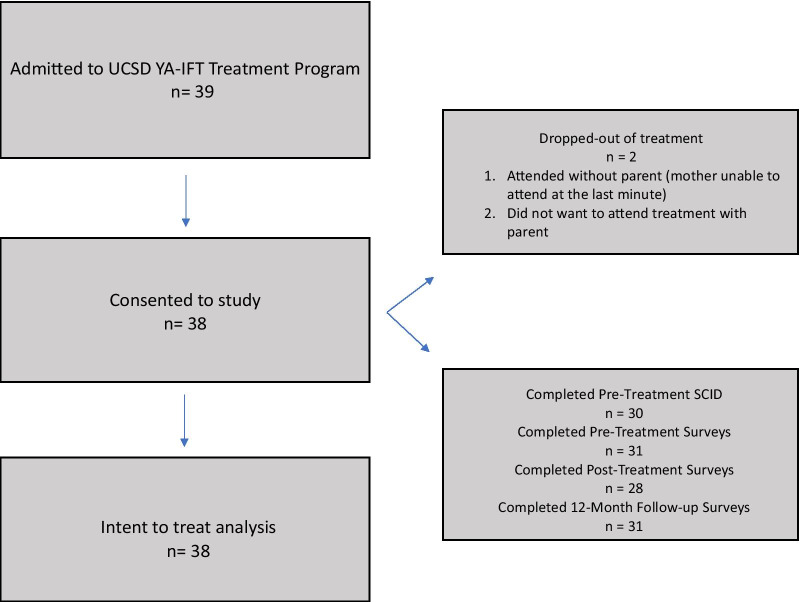
Participant Recruitment and Retention

YA-TBT-S weeks were provided approximately every quarter between October 2018 and June 2019. Each treatment week enrolled a mean of 6.33 (*SD* = 0.75) families. A total of thirty nine patients and their support(s) attended a YA-TBT-S week between October of 2017 and June of 2019, and thirty eight patients consented to participate in the study. Recuitment for the program ceased in the third or fourth quarter of 2019 due to staffing issues. In 2020, in-person YA-TBT-S was stopped due to the pandemic, and data collection on virtual YA-TBT-S began. Information on patient retention is displayed in Fig. 1. Participants who did not complete pre-treatment surveys (n = 7) did not differ on admit age, race, admit BMI, duration of illness, eating disorder diagnosis, number of current or lifetime comorbidities, or age of onset when compared to participants who completed their pre-treatment surveys (*p*s > 0.23). Participants who did not complete discharge surveys (*n* = 10) did not significantly differ from those who did complete the surveys on any of the admission variables, discharge BMI, CIA, EDE-Q, or STAI scores at admission (*p*s > 0.13). Finally, participants who were lost to follow-up (*n* = 7) did not significantly differ from participants who completed follow-up surveys on any of the admission variables, discharge BMI, CIA, EDE-Q, or STAI scores at discharge (*p*s > 0.22).

### Attrition and acceptability

Thirty-six patients completed treatment, and two patients were lost to attrition, resulting in a 5.3% attrition rate. One patient attended with a sibling and no parent and left prematurely feeling that the program did not meet her needs. The other left due to not wanting to attend treatment alongside her parents. Items assessing fundamental treatment components indicated that the patients (n = 24): liked the neurobiology exercises (*m* = 4.75/5; SD 0.53), felt better equipped with tools and more confident in their recoveries (*m* = 4.43; SD 0.66; and *m* = 4.46; SD 0.59 respectively), and had a plan for parent involvement (*m* = 4.38/5; SD 0.65). At post-treatment, 20 patients who completed acceptability questionnaires, including the Client Satisfaction Questionnaire (CSQ), reported high ratings of satisfaction with the treatment with an average rating of 3.8/4 (SD 0.47), and a transformed score of 95/100. The CSQ was added as a measure after the study commenced, resulting in a discrepancy of four participants who completed post-treatment surveys, but did not complete this questionnaire.

At post-treatment, twenty-two supports completed the CSQ and twenty seven supports completed a treatment-specific measure of acceptability. Supports reported high treatment satisfaction and acceptability, with an average CSQ rating of 3.82/4 (SD = 0.40) and an transformed score of 95.59/100. Individual items indicated supports gained a better understanding of the eating disorder through neurobiology exercises (*m* = 4.67/5; SD 0.56), felt better equipped with tools to help their loved one (*m* = 4.63/5; SD 0.49), felt more confident about their loved one’s recovery (*m* = 4.46/5; SD 0.59), felt that their parental role in recovery had been clarified (*m* = 4.21/5; SD 0.75), planned to stay involved in their loved one’s recovery (*m* = 4.93/5; SD 0.27), and felt that the behavioral contract would be effective in helping them support recovery (*m* = 4.29; SD 0.75) (Table 2).

**Table 2:** Acceptability and adherence ratings

Client Satisfaction Questionnaire (CSQ-8) Post Treatment Mean (SD) (1–4 Likert scale)	*Patients* *n* = 20	*Supports* *n* = *22*
1. How would you rate the quality of the service you have received?	4.00 (0.0)	3.95 (.21)
2. Did you get the kind of services you wanted?	3.90 (0.31)	3.86 (.35)
3. To what extent has our program met your needs?	3.70 (0.57)	3.72 (.46)
4. If a friend were in need of similar help, would you recommend our program to him or her?	3.95 (0.22)	3.95 (.21)
5. How satisfied are you with the amount of help you have received?	3.75 (0.44)	3.59 (.91)
6. Have the services you received helped you to deal more effectively with your problems?	3.60 (0.60)	3.77 (.43)
7. In an overall, general sense, how satisfied are you with the service you have received?	3.90 (0.31)	3.91 (.29)
8. If you were to seek help again, would you come back to our program?	3.75 (0.55)	3.82 (.40)
9. CSQ TOTAL	30.55 (2.01)	30.59 (1.71)
	(95.46/100)	(95.59/100)

Treatment Acceptability Ratings Post-Treatment Mean (1–5 Likert scale)	*Patients* *n* = 24	*Supports* *n* = *27*
The neurobiology exercises improved my understanding of my/my loved one’s eating disorder	4.71(0.55)	4.67 (.56)
I feel better equipped with more and better tools to use throughout my recovery/to help my loved one in their recovery	4.43 (0.66)	4.63 (.49)
I am more confident about my ability to recover/my loved one’s ability to recover	4.46 (0.59)	4.48 (.75)
I feel that my parent(s) role in my recovery/my role in my loved one’s recovery has been clarified	4.21 (0.75)	4.48 (.70)
I plan to continue to allow my parents to be involved in my recovery/to be involved in my loved one’s recovery	4.38 (0.65)	4.93 (.27)
Working on developing a contract will help me be more effective in my recovery/in supporting my loved one’s recovery	4.29 (0.75)	4.94 (.79)
I learned skills from other patients and supports that I can now apply to my recovery/my loved one’s recovery	4.50 (0.66)	4.53 (.58)

Treatment Adherence Ratings 12-Month Follow-up % Endorsed *Frequently, Almost Always, Always*	*n* = 20	*–*
Since the program, how often did you:		–
Follow the contract created?	50%	–
Follow the prescribed meal plan?	55%	–
Work with or involve your family in treatment?	75%	–
Think about or use what you learned about the neurobiology of eating disorders to help you manage your disorder?	75%	–

Client Satisfaction Questionnaire: CSQ acceptability ratings based on a 4-point Likert scale with higher ratings indicating greater satisfaction.

CSQ Total raw score was transformed to obtain a distribution out of 100 for ease of interpretation.

Treatment acceptability: Ratings based on a 5-point Likert scale with higher ratings indicating greater acceptability.

Treatment adherence: Rating based on a 5-point scale assessing frequency including *Never, Seldom, Frequently, Almost Always, Always*

Twelve-month follow-up data were not collected on supports.

### BMI

**Fig. 2:**
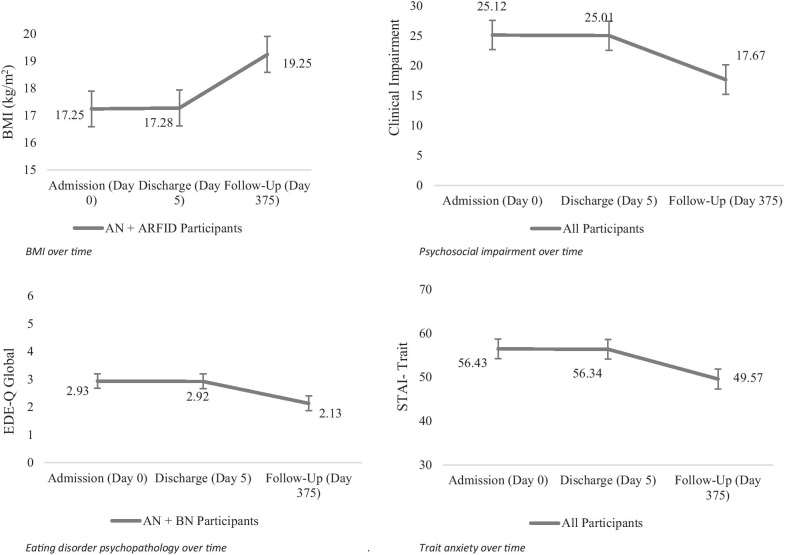
Outcomes Variables over Time. Estimated marginal means for all outcomes variables are displayed at mean levels of age and length of illness. Participants with a diagnosis of AN or ARFID and BMI under 18.5 kg/m^2^ were included in the BMI model, while participants with AN and BN were included in the EDE-Q (eating disorder psychopathology) model. All participants were included in both the STAI (trait anxiety) and CIA (psychosocial impairment) models. AN, Anorexia Nervosa; ARFID, Avoidant and Restrictive Food Intake Disorder; BN, Bulimia Nervosa

**Fig. 3:**
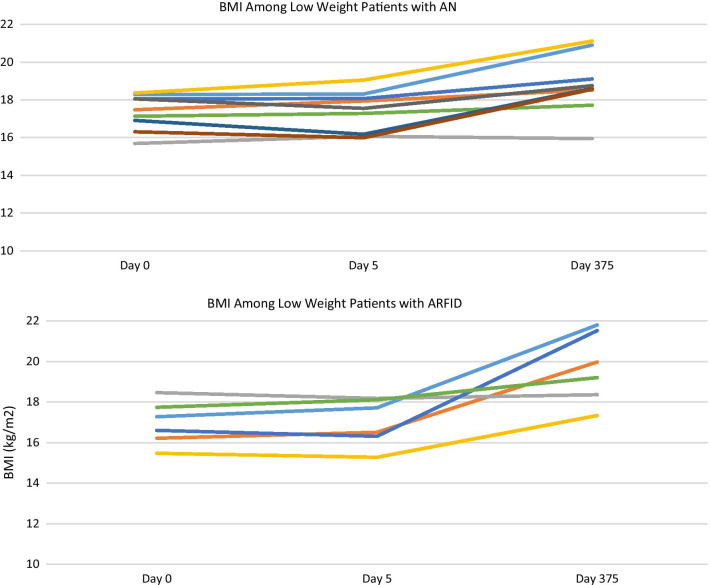
Fixed Predicted Values of Low BMI by Diagnosis. Participants with BMI less than or equal to 18.5 kg/m^2^ at admission are included in the graphs

BMI values were analyzed for participants with BMI values of ≤ 18.5 kg/m^2^ (n = 15 with AN and ARFID diagnoses). There was no significant time by week interaction (*b*s = − 0.005 to 0.006, *p* = 0.41, *r*s = 0.03–0.51), suggesting changes in BMI did not depend on the treatment week that participants attended. The main effect of time remained significant upon removing the time by week interaction term (Table 3), suggesting BMI increased over time among participants with ARFID and AN (*p* < 0.001; see Fig. 2). See Fig. 3 for more information.

**Table 3:** Multilevel model estimates of predictors over time

Predictor	BMI			EDEQ Global			CIA			STAI		
	Est	*p*	*r* (95% CI)	Est	*p*	*r* (95% CI)	Est	*p*	*r* (95% CI)	Est	*p*	*r* (95% CI)
Intercept	17.07	< .001*	.99 (.98–.99)	2.81	.001*	.56 (.23–.71)	18.72	< .001*	.52 (.19–.69)	53.53	< .001*	.84 (.71–.91)
Time	.01	< .001*	.81 (.66–.90)	− .002	.002*	.45 (.15–.67)	− .02	.002*	.42 (.13–.66)	− .02	< .001	.50 (.21–.71)
Age	− .55	< .001*	.79 (.63–.89)	− .05	.73	.06 (− .26 to .37)	− .59	.53	.13 (− .20 to .43)	.87	.34	.13 (− .20 to .43)
Length of Illness	.01	.03*	.54 (.19–.69)	− .002	.99	.02 (− .30 to .33)	− .05	.19	.15 (− .18 to .45)	− .07	.08	.23 (− .10 to .51)
YA-TBT-S Week 1	1.03	.15	.36 (.05–.61)	.01	.99	.01 (− .31 to .33)	8.03	.23	.15(− .18 to .45)	2.79	.65	.06 (− .26 to .37)
YA-TBT-S Week 2	.87	.11	.40 (.09–.64)	.56	.59	.09 (− .24 to .24)	12.88	.05	.34 (.02–.60)	8.44	.20	.22 (− .11 to .50)
YA-TBT-S Week 3	.03	.95	.02 (− .30 to .34)	.13	.90	.02 (− .30 to .33)	9.27	.14	.27 (− .05 to .54)	6.21	.31	.18 (− .15 to .47)
YA-TBT-S Week 4	− 1.01	.08	.44 (.33–.07)	− .76	.48	.13 (− .20 to .43)	− .61	.92	.04 (− .28 to .36)	− 4.55	.47	.15 (− .18 to .45)
YA_TBT-S Week 5	− 1.51	.11	.60 (.35–.77)	.82	.43	.02 (− .30 to .33)	7.91	.21	.19 (− .14 to .48)	4.34	.48	.11 (− .22 to .42)
YA-TBT-S Week 6	–	–	–	–	–	–	–	–	–	–	–	–

The time by week interaction term was not a significant predictor of change in outcomes over time and was subsequently removed from all models. As such, it is not displayed in the table of estimates. The sixth YA-TBT-S week was the referent week in all models

Est., estimate; CI, confidence interval; YA-TBT-S, Young Adult Temperament Based Treatment with Supports

### EDE-Q global score

**Fig. 4:**
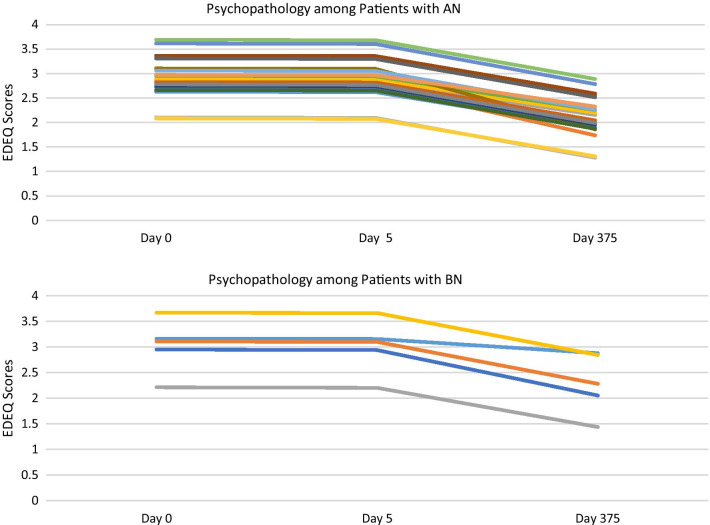
Fixed Predicted Values of Eating Disorder Psychopathology by Diagnosis

There was no significant time by week (*b*s = − 0.89 to 0.98, *p* = 0.80, *r*s = 0.01–0.08) interaction, suggesting changes in EDE-Q global scores did not depend) on the treatment week attended. Upon removing the interaction term, there was a significant effect of time (Table 3), such that EDE-Q scores improved over time among participants with AN and BN (Fig. 2). See Fig. 4 to view EDE-Q trajectories.

### Binge eating & purging

Participants with AN-BP and BN reported engaging in an average of 14.50 (*n* = 12, *SD* = 16.85) binge eating episodes in the 28 days prior to admission, 5.2 (*n* = 10, *SD* = 8.85) binge eating episodes at post-treatment (assessed over a seven day time period and transformed to be consistent with the 28 day period by multiplying by four), and 7.2 (*n* = 10, *SD* = 8.78) binge eating episodes in the 28 days prior to 12-month follow-up. They reported a mean of 25.17 (*n* = 12, *SD* = 32.22) purging episodes (i.e., self-induced vomiting or laxative abuse) at admission, 4.4 (*n* = 10, *SD* = 10.06) at discharge, and 7 (*n* = 10, *SD* = 8.52) at follow-up.

### Clinical impairment assessment

**Fig. 5:**
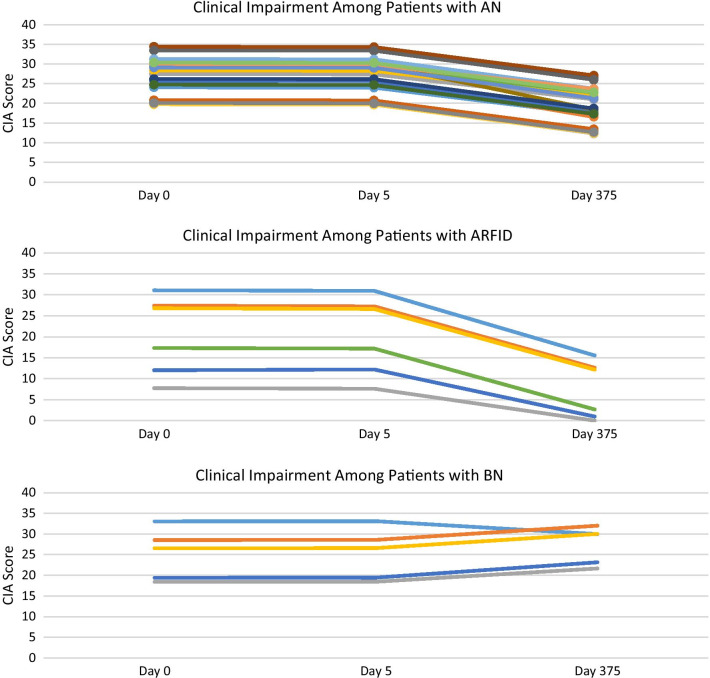
Fixed Predicted Values of Clinical Impairment by Diagnosis

There was no significant time by week interaction (*b*s = − 0.02 to 0.01, *p* = 0.14, *r*s = 0.07–0.18), suggesting changes in CIA scores did not depend on the treatment week in which participants attended. Upon removing the non-significant interaction term, there was a main effect of time (Table 3), such that clinical impairment decreased over time across participants (Fig. 2). See Fig. 5 for individual trajectories of clinical impairment.

### Trait anxiety

**Fig. 6:**
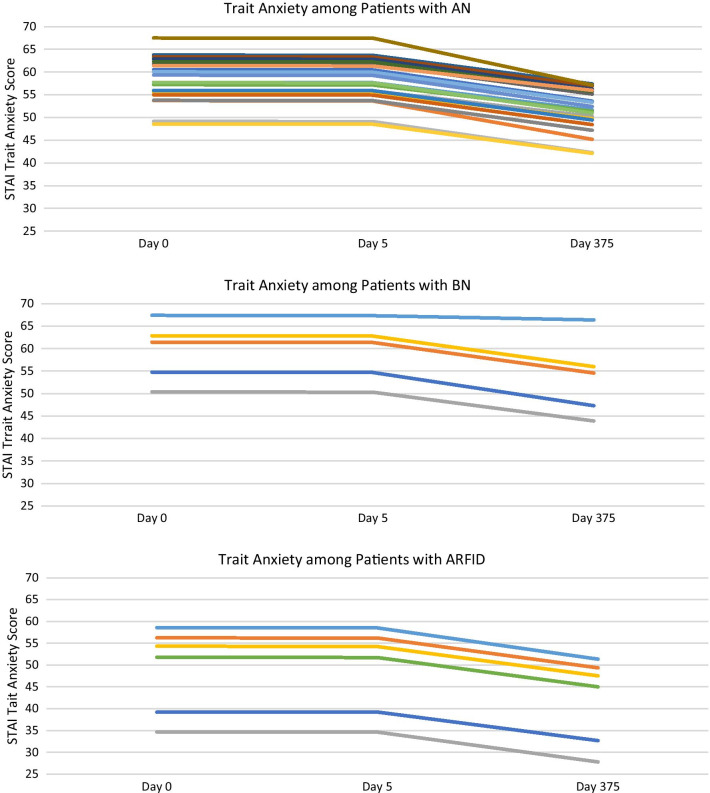
Fixed Predicted Values of Trait Anxiety by Diagnosis

There was no significant time by week (*b*s = − 0.07 to 0.02, *p* = 0.19, r*s* = 0.04–0.22) interaction, suggesting changes in trait anxiety did not depend on the treatment week attended. Upon removing the non-significant interaction term, there was a significant effect of time (Table 3), such that trait anxiety decreased over time across participants (Fig. 2). See Fig. 6 for more information.

### Remission rates

At admission, 12.12% of participants were classified as fully (*n* = 2, 6.06%) or partially (*n* = 2, 6.06%) remitted on the basis of being within a normative weight range. The remainder of participants (*n* = 29, 87.88%) were classified as ill (i.e., not remitted) upon admitting to treatment. Specifically, 14 participants were regularly bingeing and/or purging, 13 participants had a low BMI, and two participants had both a low BMI and were regularly bingeing and/or purging. At discharge, 34.38% of participants were classified as fully (n = 5, 15.63%) or partially (*n* = 6, 18.75%) remitted. Twenty-one participants (65.63%) reported a poor outcome at discharge (14 due to low BMI, 6 due to bingeing and/or purging, and 1 due to both low BMI and purging). At follow-up, 53.33% of participants were classified as fully (*n* = 13; 43.33%) or partially remitted (*n* = 3; 10%), while 46.67% reported a poor outcome (*n* = 14). Of the 14 participants with a poor outcome, nine reported bingeing and/or purging, four reported a low BMI, and one reported both low BMI and bingeing and purging. One participant could not be classified at follow-up due to not knowing her weight. Across diagnoses, the proportion of participants with a good outcome (i.e., classified as fully or partially remitted) was significantly improved from admission to follow-up (*p* < 0.001) but not from admission to discharge (*p* = 0.06) or discharge to follow-up (*p* = 0.09). Remission rates by diagnosis are presented in Table 4.

**Table 4:** Remission rates by diagnosis

	Full Remission			Partial Remission		
	Admission	Discharge	Follow-Up	Admission	Discharge	Follow-Up
	n(%)	n(%)	n(%)	n(%)	n(%)	n(%)
AN	2/20 (10%)	4/21 (20%)	7/20 (35%)	2/20 (10%)	6/21 (28.57%)	3/20 (15%)
BN	0/7 (0%)	1/5 (20%)	1/4 (25%)	0/7 (0%)	0/5 (0%)	0/4 (0%)
ARFID	0/6 (0%)	0/6 (0%)	5/6 (83.33%)	-	-	-
Total	2/33 (6.06%)	5/32 (15.63%)	13/30 (43.33%)	2/27 (7.41%)	6/26 (23.08%)	3/24 (12.5%)

AN, Anorexia Nervosa; BN, Bulimia Nervosa; ARFID, Avoidant Restrictive Food Intake Disorder

Full remission for AN and BN were defined as BMI > 18.5 kg/m^2^, no bingeing or purging for the past 28 days, and an EDEQ Global score < 2.91. Partial remission criteria were the same as full remission criteria with the exception of EDEQ Global score > 2.91. Full remission from ARFID was defined as BMI > 18.5 kg/m^2^ and a CIA score < 16

#### Other treatments

No participants engaged in other concurrent treatments during program participation. Of the twenty-four participants who completed surveys at follow-up assessing treatments received between discharge and the follow-up period, 54% (n = 13) reported some form of outpatient care, 29.2% (n = 7) reported participation in a partial hospitalization program, and 8.3% (n = 2) reported an inpatient hospitalization stay compared to 15.7% (n = 8) prior to admission.

## Discussion

The present study examined the acceptability and treatment outcomes for thirty-eight ED patients who attended an intensive, multi-family treatment program for young adults with EDs and their parent-supports. The YA-TBT-S program delivers a Temperament-Based Treatment approach (TBT-S) and a parent-supported model of treatment specifically designed for emerging adults. Temperament-Based Treatment with Supports (TBT-S) is a novel treatment approach that targets EDs based on underlying temperament and neurobiology using psychoeducation and skills training. The version of TBT-S evaluated in this study includes specific adaptations for emerging adults, including a novel model of parental support delivered over a five-day intensive, multi-family format.

Participants were patients with AN-spectrum, BN-spectrum, and ARFID diagnoses who completed assessments at pre-treatment, post-treatment, and approximately 12-months after completing the treatment program. Consistent with previous studies evaluating TBT-S, the treatment was feasible, had low attrition, and high acceptability [37]. The findings from this study indicate that YA-TBT-S may be a promising adjunctive treatment for young adults with a range of EDs, and demonstrate that applying a parent-involved model to young adults with EDs is feasible and acceptable. The attrition rate was 5.3%, which compares favorably to other ED treatments [11, 15, 17]. Favorable ratings were also endorsed for aspects of treatment that are novel to our treatment model, with a significant portion of participants reporting continued use of these methods suggesting that these treatment strategies are durable over time.

Remission rates significantly improved from admission to follow-up. By follow-up, 53.33% of the sample was classified as fully or partially recovered. Additionally, underweight patients with AN and ARFID showed statistically and clinically significant increases in BMI, with a gain of approximately 2 BMI points from admission to follow-up. This BMI increase is comparable to BMI increases reported in other AN treatment outcome studies [23]. There were also significant reductions in participants’ psychopathology as measured by the EDE-Q (for AN and BN) and the CIA. The reductions in psychopathology are clinically notable, however a significant portion of YA-TBT-S participants received other treatments between discharge and follow-up, and thus we are unable to determine the specific effect of TBT-S on these outcomes.

Outcomes across time were examined for individual cases to study trends in each diagnostic group (See Figs. 3, 4 and 5). Compared to those with AN and ARFID, the results for patients with BN were mixed. While only four participants with BN (50%) were retained at follow-up, just one participant reported a good outcome (i.e., full remission). The remaining three were classified as non-responders due to ongoing bingeing and purging. Participants with BN demonstrated a trend towards reductions in ED psychopathology over time, however their clinical impairment scores did not improve. Due to our sample size and behavior frequency, we were not able to perform analyses to determine the significance of changes in binge and purge behaviors over time. There was a mean reduction in binge-purge behaviors at discharge and follow-up among participants with BN, however participants continued to report binge and purge behaviors. These results suggest that YA-TBT-S may not be suited for individuals with BN. This may be related to the emphasis in TBT-S on strategies informed by temperament and neurobiological mechanisms that more closely align with restrictive eating disorders. However, our BN sample consisted of only eight participants, and more research is needed to determine the suitability for this population.

Taken together, results support the feasibility and acceptability of YA-TBT-S for young adults with eating disorders. Preliminary outcomes for program participants show changes in core pathology over a 12-month period, although we are unable to attribute favorable outcomes to participation in the program due to other treatment received within the follow-up period. Given that a significant portion of participants received other treatments upon discharge, YA-TBT-S may be best construed as a adjunctive treatment to bolster family involvement.

Notably, there was a significant decrease in trait anxiety over time across diagnoses, however trait anxiety scores remained elevated at the 12-month follow up period. The small effect of treatment on trait anxiety may be consistent with the TBT-S treatment philosophy, where participants are taught that trait anxiety is stable and consistent over the lifetime. Instead, the treatment emphasizes minimizing the effects of anxiety on eating behaviors versus reducing general trait anxiety.

This study has several important strengths. YA-TBT-S is a novel treatment model for a range of EDs which may hold promise as an adjunctive treatment in treating subsets of ED populations where currently available treatments continue to be limited or under-accessed, or parent involvement may be helpful or necessary. Notably, the treatment was rated highly amongst both patient and parent participants. An approach that is tailored for developmental stage and which leverages parent-support may have the potential to improve treatment access for those with a range of illnesses and severity levels. This is critical given studies suggesting the prevalence and persistence of disordered eating behavior amongst young adults. Although the study sample may be characterized as less severe based on BMI status, we consider the broad catchment of illness severity a strength given studies pointing to the low treatment utilization rates for less severe EDs, and associated risks with later pathology [9, 10, 31]. This study also possesses strong external validity due to the naturalistic design in a real-world clinic setting. Other strengths of this study include a year-long follow-up period, which is difficult to attain in a naturalistic study, the use of sound psychometric measures, and the use of maximum likelihood estimation to handle missing data.

There are also several important limitations to note. First, due to the brief nature of treatment (five days), the majority of participants received follow-up care upon discharge. The continuity of other treatment could have contributed to outcomes at the 12-month follow-up period and prevents us from determining the effect of the YA-TBT-S program on outcomes. Notably, treatment adherence ratings suggest that a substantial portion of participants continued to use prescribed TBT-S-specific treatment methods at the 12-month follow-up period. Due to the brief course of treatment inherent in our model, recommendations for follow-up treatment are considered an important treatment objective. In addition to being a closed treatment program, patients and parent supports are introduced to the intensive program as a means to assess continued treatment needs. YA-TBT-S may be best framed as an intensive, introductory treatment primer that may increase the uptake and access to more traditional, specialized treatments. Future studies should also examine potential outcome mediators to elucidate potent aspects of treatment since the treatment included a number of therapeutic variables such as multi-family treatment, parent involvement, neurobiology, and skills training.

The study is also limited by the small sample size and missing data. An approximated power analysis suggests that our study was underpowered, which may not have allowed us to detect important differences and limited our ability to conduct post-hoc analyses. Further, remission rates classifications at discharge include modifications made to the EDE-Q and the CIA to account for a seven day time-period (versus 28 days), and the lack of standardized classification criteria for ARFID patients. Lastly, this study relied on self-report surveys to assess outcomes.

## Conclusions

This naturalistic study of YA-TBT-S introduces a novel program for young adults and their parent-supports. Findings from this study point to promising outcomes for those who receive YA-TBT-S as an adjunct to traditional modes of care. This finding is of relevance given the current treatment limitations for adults with EDs and the lack of specialized treatments for YA. Future studies are needed including large, well-powered randomized controlled trials to determine treatment efficacy compared to other proven treatments for young adults with EDs.

## Data Availability

Written requests are required for dataset and will be granted upon reasonable request.

## Abbreviations

ED: Eating disorders
YA: Young adults
AN: Anorexia nervosa
BN: Bulimia nervosa
ARFID: Avoidant/restrictive food intake disorder
BMI: Body mass index
